# Supplementary material for: Late Pleistocene South American megafaunal extinctions associated with rise of Fishtail points and human population
Source: Nat Commun. 2021 Apr 12;12:2175. doi: 10.1038/s41467-021-22506-4 (PMC8041891; doi:10.1038/s41467-021-22506-4)
Supplement: Supplementary file 1 — Supplementary Information [file 41467_2021_22506_MOESM1_ESM.pdf]

Supplementary Information

Late Pleistocene South American megafaunal extinctions associated with rise of Fishtail points and human population

Luciano Prates and S. Ivan Perez

Supplementary Figures

Figure S1. Map of the studied sites and regions. Dates on large mammals are represented by diamonds and dates on human archaeological evidence by dots. Colors of the symbols represent regions: Andes (light blue), Pampa and South East Brazil (light red) and Patagonia (light green).

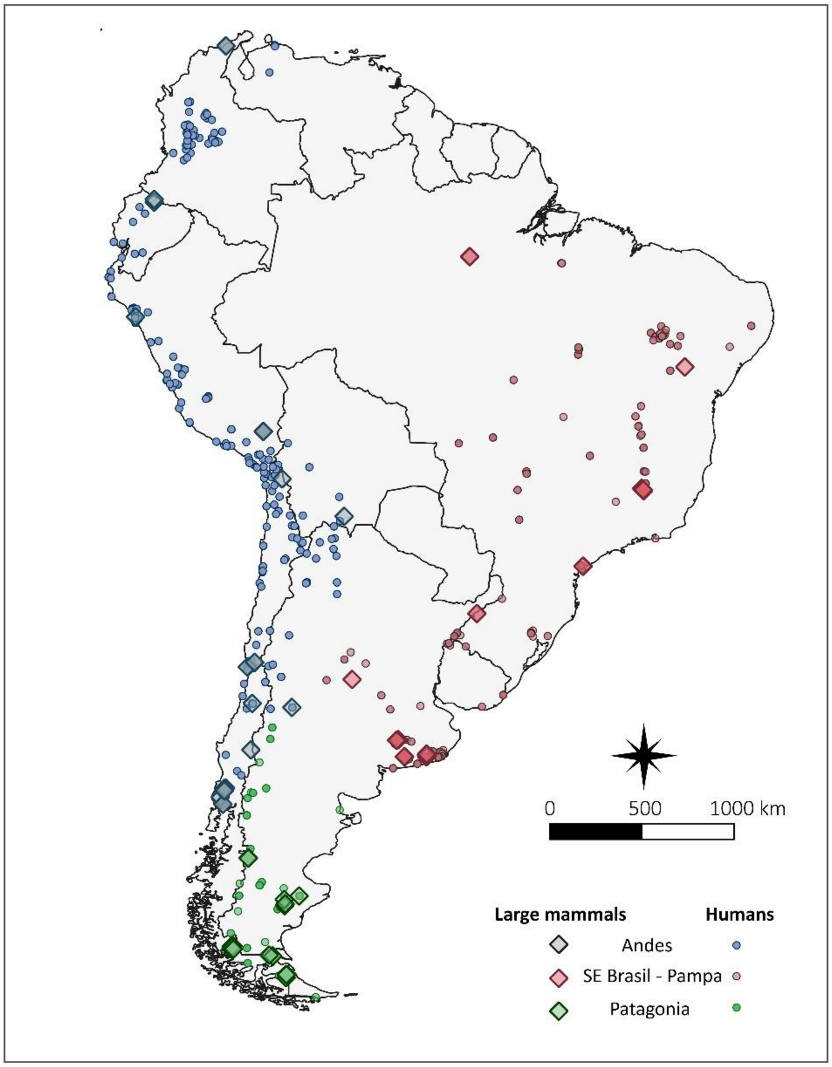

Figure S2. Temporal changes in the density of each large mammal species from South America. Gray shading areas represent lower and upper bounds of the simulated envelope, and black lines show observed summed probability curves. Red and blue colors represent positive and negative deviation from global trend, respectively.

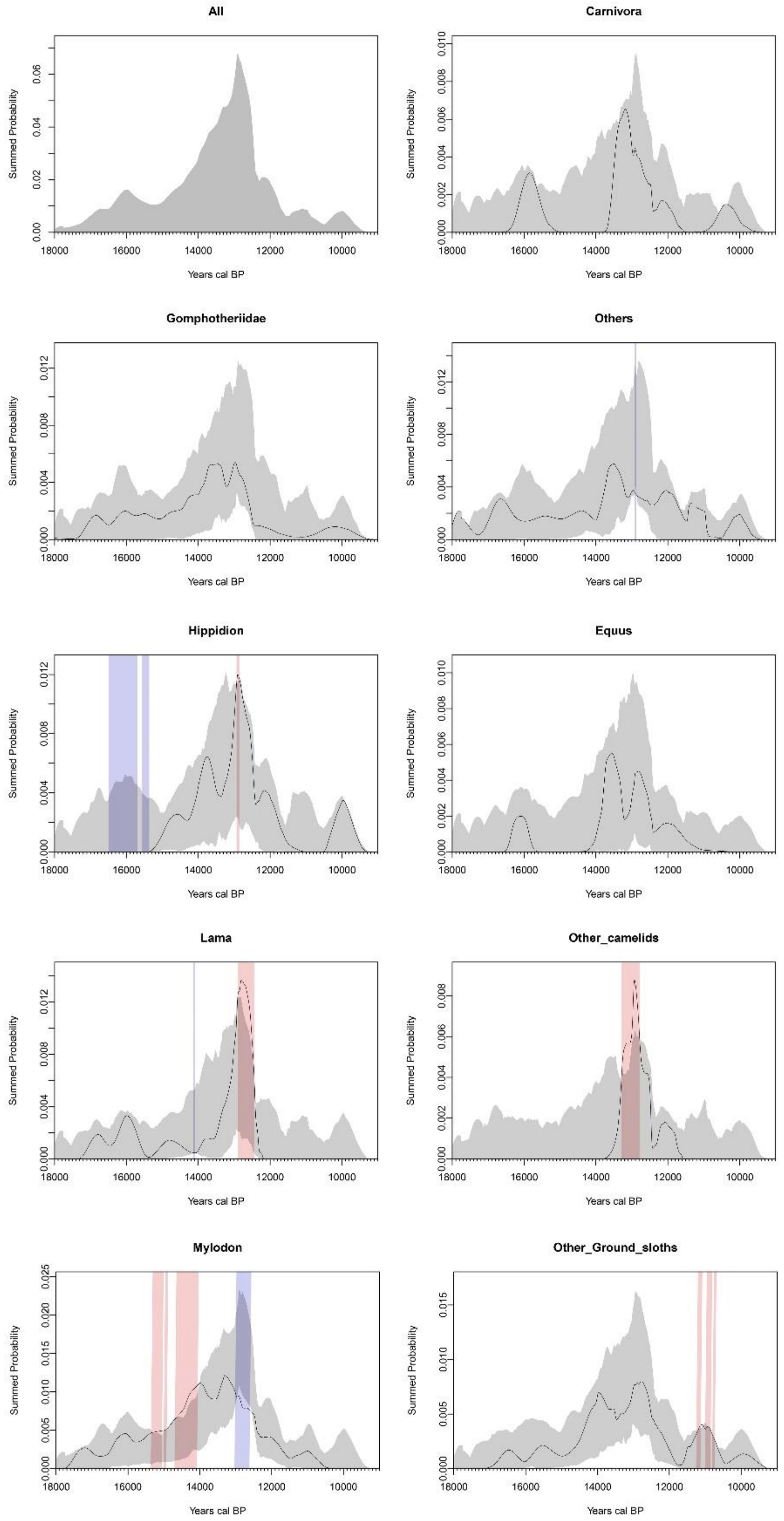

Figure S3. Kernel density of Fishtail projectile points from South America. Density of Fishtail projectile points varies from low (blue) to high (red).

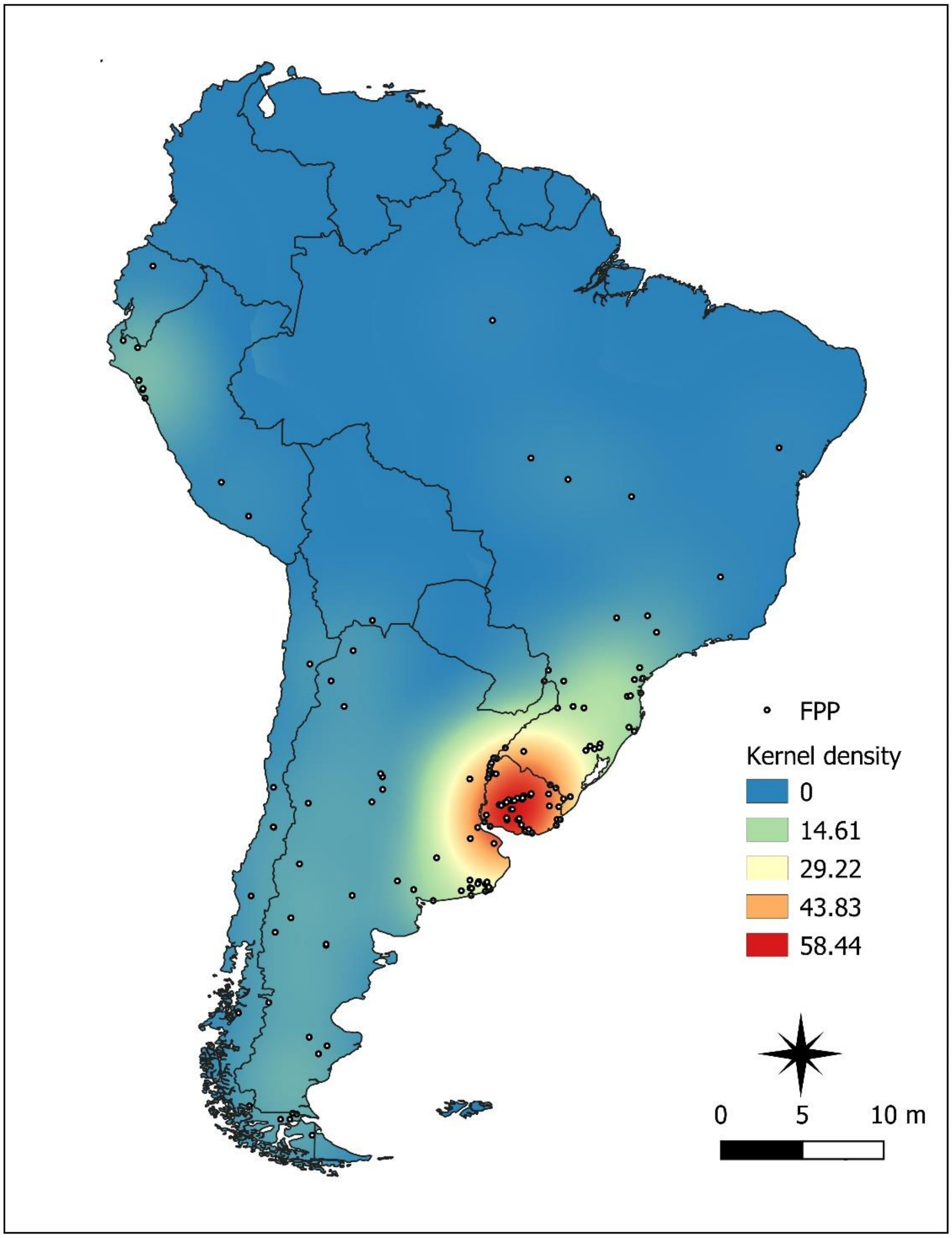

Figure S4. Kernel density of all archaeological sites from South America for different periods (15,5 k – 13,3 k, 13,3 k – 11 k, and 11 k – 9 k cal BP). Density of sites varies from low (blue) to high (red).

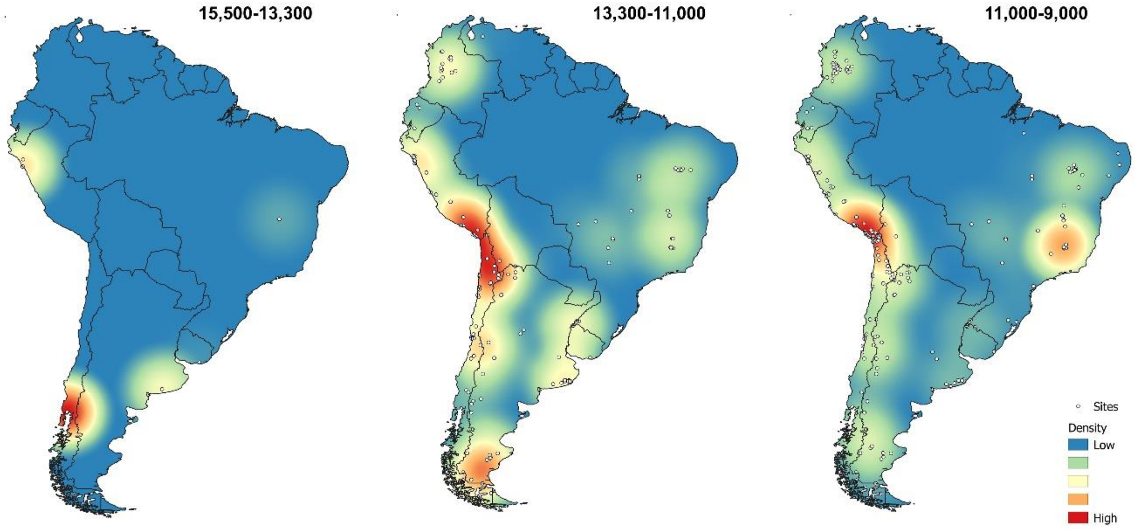

Supplementary Tables

Supplementary Table 1

a

	All-Sites	FPP	*C. hydon*	*E. neogeus*	*M. darwini*	*D. clavicaudatus*	*G. robustum*	*H. saldiasi*	*N. platensis*	*L. gracilis*	*M. americanus*	*N. waringi*
All-Sites	1,000000	0,849198	0,806273	0,743118	0,379401	0,450619	0,462773	0,467691	0,566153	0,649204	0,430546	0,632626
FPP	0,849198	1,000000	0,601525	0,772494	0,403414	0,717442	0,725939	0,472647	0,778321	0,811496	0,674030	0,425978
C. hydon	0,806273	0,601525	1,000000	0,619434	0,118601	0,230858	0,260564	0,211977	0,311521	0,349043	0,240798	0,838667
E. neogeus	0,743118	0,772494	0,619434	1,000000	0,079880	0,670220	0,721100	0,100997	0,731653	0,545784	0,679096	0,537658
M. darwini	0,379401	0,403414	0,118601	0,079880	1,000000	0,198049	0,082741	0,870083	0,201883	0,618469	0,101753	0,126886
D. clavicaudatus	0,450619	0,717442	0,230858	0,670220	0,198049	1,000000	0,886322	0,231081	0,830518	0,754911	0,909449	0,115243
G. robustum	0,462773	0,725939	0,260564	0,721100	0,082741	0,886322	1,000000	0,094977	0,853162	0,613725	0,946052	0,121228
H. saldiasi	0,467691	0,472647	0,211977	0,100997	0,870083	0,231081	0,094977	1,000000	0,190067	0,712980	0,118208	0,127919
N. platensis	0,566153	0,778321	0,311521	0,731653	0,201883	0,830518	0,853162	0,190067	1,000000	0,679095	0,808475	0,163513
L. gracilis	0,649204	0,811496	0,349043	0,545784	0,618469	0,754911	0,613725	0,712980	0,679095	1,000000	0,630406	0,188678
M. americanus	0,430546	0,674030	0,240798	0,679096	0,101753	0,909449	0,946052	0,118208	0,808475	0,630406	1,000000	0,114791
N. waringi	0,632626	0,425978	0,838667	0,537658	0,126886	0,115243	0,121228	0,127919	0,163513	0,188678	0,114791	1,000000

b

Name	Axis 1	Axis 2
All-Sites	0,10489	0,08693
FPP	0,01150	0,00112
C. hydon	0,15582	0,31341
E. neogeus	-0,13675	0,18582
M. darwini	0,31801	-0,26598
D. clavicaudatus	-0,23710	-0,09771

G. robustum	-0,27151	-0,04553
H. saldiasi	0,32455	-0,24000
N. platensis	-0,21729	-0,03916
L. gracilis	0,01513	-0,18827
M. americanum	-0,27436	-0,05980
N. waringi	0,20713	0,34917

Stress	0,0642
R2 - Dim 1	0,5558
R2 - Dim 2	0,5281

Supplementary Table 1. Results of niche overlapping analyses. a) Matrix of I similarity statistic describing the values of niche overlapping among megafaunal species and FFP distribution. b) nm-MDS analysis details.
