# Supplementary material for: Late Pleistocene South American megafaunal extinctions associated with rise of Fishtail points and human population
Source: Nat Commun. 2021 Apr 12;12:2175. doi: 10.1038/s41467-021-22506-4 (PMC8041891; doi:10.1038/s41467-021-22506-4)
Supplement: Supplementary file 3 — Description of Additional Supplementary Files [file 41467_2021_22506_MOESM3_ESM.pdf]

Description of Additional Supplementary Files

File Name: Supplementary Data 1
Description: Radiocarbon dates for South American Fishtail projectile point records.

File Name: Supplementary Data 2
Description: Radiocarbon dates for South American megafaunal records.

File Name: Supplementary Data 3
Description: Geographical coordinates for South American Fishtail projectile point records.

File Name: Supplementary Data 4
Description: Geographical coordinates for South American megafaunal records.

File Name: Supplementary Code 1
Description: R scripts to analyze data
