# Supplementary material for: Late Pleistocene South American megafaunal extinctions associated with rise of Fishtail points and human population
Source: Nat Commun. 2021 Apr 12;12:2175. doi: 10.1038/s41467-021-22506-4 (PMC8041891; doi:10.1038/s41467-021-22506-4)
Supplement: Supplementary file 8 — Supplementary Code 1 [file 41467_2021_22506_MOESM8_ESM.pdf]

Supplementary Code 1

Late Pleistocene South American megafaunal extinctions associated with rise of Fishtail points and human population

Luciano Prates and S. Ivan Perez

##The data for running temporal and spatial analyses in R packages are provided in the Supplementary Data 1 to 4

##R script for analyzing radiocarbon dates in *rcarbon* package

```
require(rcarbon)

#Open radiocarbon data - example
nqn<-read.csv("FPP.csv", h=T)
nqn
attach(nqn)

#Radiocarbon data preparation
bins <- binPrep(Site,C14Age,h=200)
bins

#Calibrate radiocarbon data for SPD
x <- calibrate(C14Age,C14SD, calCurves='shcal20')
x

#Calculate empirical SPD
spd.nqn5 <- spd(x,bins=bins, runm=500,timeRange=c(14000,10500))
plot(spd.nqn5)

#Comparing empirical SPDs against each other
perm.NPat=permTest(x=x,marks=as.character(Region),
timeRange=c(14000,10500),bins=bins,nsim=100,runm=500)

#Ploting results
par(mfrow=c(3,1))
plot(spd.nqn5, main="Global")
plot(perm.NPat, focalm = "Andes", main="Andes")
plot(perm.NPat, focalm = "Pampa", main="Pampa")
plot(perm.NPat, focalm = "Patagonia ", main="Patagonia")
```

##R script for analyzing occurrence data in *SSDM* package

```
require (dismo)
require (SSDM)
# only run if the maxent.jar file is available, in the right folder
jar <- paste(system.file(package="dismo"), "/java/maxent.jar", sep='')

###Read files
# File with presence points or occurrence data
occ <- read.table("TodosGenerosFinal.csv", header=TRUE, sep=',')

occ2<-load_occ(path = getwd(), predictors, Xcol = 'LONGITUDE', Ycol = 'LATITUDE', Spcol = 'SPECIES',  file = 'TodosGenerosFinal.csv', sep = ',')

occ2

# Get predictor variables. All .GTiff images obtained from http://www.paleoclim.org/ must to be available in the same folder than the .csv file. We crop the GTiff images in QGIS 3.14 using a South American shapefile

fnames <- list.files (pattern='tif', full.names=TRUE)
fnames

predictors <- stack(fnames)
plot(predictors)


# Stacked distribution (SSDM) building
SSDM2 <- stack_modelling('MAXENT', occ2, predictors, rep = 1,
                         Xcol = 'LONGITUDE', Ycol = 'LATITUDE',
                         Spcol = 'SPECIES', cores = 6)

# SSDM results plotting
plot(SSDM2)

# Saving SDM results
save.stack(SSDM2, name = strsplit(SSDM2@name, ".Stacked2.SDM", fixed = TRUE)[[1]][1], path = getwd(), verbose = TRUE, GUI = FALSE)
```

##R script for estimating niche overlap index

```
#Example niche overlaping. The raster files in GTiff format are saving or obtaining from the stack_modelling estimation

arP <- writeRaster(rP, filename="MaxentPuntasocc.tif", format="GTiff", overwrite=TRUE)
arH <- writeRaster(r, filename="MaxentMegatheriumFinal_new.tif", format="GTiff", overwrite=TRUE)
```

```
nicheOverlap(rP, rH, stat = "I")
```
